# A roadmap for research in octoploid strawberry

**DOI:** 10.1038/s41438-020-0252-1

**Published:** 2020-03-15

**Authors:** Vance M. Whitaker, Steven J. Knapp, Michael A. Hardigan, Patrick P. Edger, Janet P. Slovin, Nahla V. Bassil, Timo Hytönen, Kathryn K. Mackenzie, Seonghee Lee, Sook Jung, Dorrie Main, Christopher R. Barbey, Sujeet Verma

**Affiliations:** 1 University of Florida, Institute of Food and Agricultural Sciences, Gulf Coast Research and Education Center, Wimauma, Florida 33598 USA; 2 Department of Plant Sciences, University of California, Davis, CA 95616 USA; 3 Department of Horticulture, Michigan State University, East Lansing, MI 48824 USA; 4 USDA-ARS Genetic Improvement of Fruits and Vegetables Laboratory, Beltsville, MA 20705 USA; 5 USDA-ARS National Clonal Germplasm Repository, Corvallis, OR 97333 USA; 6 Department of Agricultural Sciences, Viikki Plant Science Centre, University of Helsinki, Helsinki, 00790 Finland; 7 Organismal and Evolutionary Biology Research Programme, Faculty of Biological and Environmental Sciences, Viikki Plant Science Centre, University of Helsinki, Helsinki, 00790 Finland; 8 NIAB EMR, Kent, ME19 6BJ UK; 9 Department of Horticulture, Washington State University, Pullman, WA 99164 USA

**Keywords:** Plant breeding, Genome evolution, Agricultural genetics

## Abstract

The cultivated strawberry (*Fragaria* × *ananassa*) is an allo-octoploid species, originating nearly 300 years ago from wild progenitors from the Americas. Since that time the strawberry has become the most widely cultivated fruit crop in the world, universally appealing due to its sensory qualities and health benefits. The recent publication of the first high-quality chromosome-scale octoploid strawberry genome (cv. Camarosa) is enabling rapid advances in genetics, stimulating scientific debate and provoking new research questions. In this forward-looking review we propose avenues of research toward new biological insights and applications to agriculture. Among these are the origins of the genome, characterization of genetic variants, and big data approaches to breeding. Key areas of research in molecular biology will include the control of flowering, fruit development, fruit quality, and plant–pathogen interactions. In order to realize this potential as a global community, investments in genome resources must be continually augmented.

## Origin and organization of the octoploid strawberry genome

The earliest cultivars of allo-octoploid (2*n* = 8× = 56) garden strawberry (*Fragaria* × *ananassa* Duchesne ex Rozier) originated approximately 300 years ago from spontaneous hybrids between ecotypes of non-sympatric wild octoploid species: *Fragaria* *chiloensis* subsp. *chiloensis* from South America and *Fragaria* *virginiana* subsp. *virginiana* from North America^1–5^. Several additional wild octoploid subspecies have since been used as parents in breeding, creating an admixed population of *F*. × *ananassa* individuals with genomes that are mosaics of phylogenetically and demographically diverse progenitor genomes^4,6–13^.

The origin of octoploid strawberry has been intensely studied and widely debated^10,14–21^. While several subgenome origin hypotheses have emerged from cytogenetic, phylogenetic, and comparative genetic mapping studies^6,7,9,15,22–25^, a complete hypothesis for the origin and evolution of the octoploid genome was only recently proposed with the publication of the “Camarosa” reference genome^10^. Through phylogenetic analyses of the transcriptomes of all described extant diploid species, including four subspecies of *Fragaria vesca*, the putative subgenome donors found in the octoploid were identified as *F. vesca* subsp. *bracteata*, *Fragaria iinumae*, *Fragaria viridis*, and *Fragaria nipponica*^10^.

Edger et al.^10^ provided strong support for earlier hypotheses that *F*. *vesca* and *F*. *iinumae* were two of the four subgenome donors^7,9,19,24,26^. Until the octoploid reference genome was published, the origin of the other diploid subgenome donors had remained unclear, although multiple hypotheses had been proposed^7,9,26^. Liston et al.^20^ then reasoned that Edger et al.^10^ may have misidentified two of the progenitors due to bias from excluding in-paralogs in their phylogenetic analyses. To address this concern, Edger et al.^21^ developed a chromosome-scale assembly of the *F. iinumae* genome and reanalyzed the original data with in-paralogs. The revised analysis supported the original model that the genome of octoploid strawberry originated through successive stages of polyploidization involving four progenitor species: diploid × diploid (*F. nipponica* × *F. innumae*) → tetraploid × diploid (tetraploid ancestor × *F. viridis*) → hexaploid × diploid (hexaploid ancestor × *F. vesca* subsp. *bracteata*) → octoploid ancestor^10^.

In addition, the chromosome-scale genome assembly showed that the diploid subgenomes were not static building blocks (e.g., A–D) walled off from one another. Rather they have dynamically evolved through homoeologous exchanges, which are well-known in neopolyploids^27–29^. Homoeologous exchanges in octoploid strawberry were found to be highly biased toward the *F. vesca* subsp. *bracteata* subgenome replacing substantial portions of the other subgenomes^10^. However, homoeologous exchanges are not unidirectional. Although the chromosomes are architectural mosaics of the four diploid subgenome donors and their octoploid descendants, *F*. × *ananassa* is strongly allo-octoploid^6,9,14,22,23^. Because the *F*. × *ananassa* chromosomes are complex admixtures of genes with different phylogenetic histories via homoeologous exchanges^10,11,30^, Edger et al.^10^ developed a nomenclature that precludes oversimplified one-to-one assignments to a specific diploid progenitor.

The *F*. × *ananassa* genome has not only been reshaped by polyploidization events, especially homeologous exchanges, gene-conversion, and selection (e.g., subgenome dominance), but by repeated interspecific hybridization in breeding that has resulted in the introgression of alleles from phylogenetically and demographically diverse *F. chiloensis* and *F. virginiana* ecotypes^2,4,6–8,10,11,19^. At this point in time, the decades long debate among geneticists and evolutionary biologists about the origin of the *F*. × *ananassa* genome^7,9,16,19,31^ seems to have reached an initial zenith. Remaining disagreements might only be settled when chromosome-scale assemblies of the other hypothesized diploid progenitors (*F. nipponica* and *F. viridis*) are assembled and analyzed.

Aside from the question of subgenome origin, what other evolutionary questions might be worthy of exploration at this juncture? First, while the four extant relatives of the diploid progenitors have been putatively identified, the history and timing of the intermediate polyploids remain poorly understood. When and where were the tetraploid and hexaploid ancestors formed? Are any of the known wild polyploids endemic to Asia descendants from these intermediate polyploids? Which subgenome is dominant in these polyploids? Second, a single dominant subgenome was uncovered in *Fragaria* *×* *ananassa* that controls many important traits including fruit quality^10^. Just how deterministic is subgenome dominance? In other words, is it possible to resynthesize the octoploid with a different degree of subgenome dominance, or with a different subgenome becoming dominant? The answer to this question could have implications for genetic improvement of the cultivated species.

## Whole-genome genotyping and genetic mapping

Genotyping advances in strawberry have naturally followed advances in humans, model organisms and row crops. The development of the Affymetrix Axiom® iStraw90 single-nucleotide polymophism (SNP) genotyping array was a significant advance that enabled the facile production and exchange of genotypic information across laboratories with high reliability, minor amounts of missing data, and negligible genotyping errors^31–33^. The ease-of-use, speed of analysis, simplicity of data management, and outstanding reproducibility of SNP genotyping arrays have been important factors in their continued adoption in strawberry and other plant species with complex genomes^11,31,32,34,35^.

Underlying computational challenges associated with genotyping by sequencing (GBS) and other next-generation sequencing (NGS) facilitated approaches have limited their widespread application in octoploid strawberry thus far^36,37^. The challenges are similar across species, but obviously exacerbated in allogamous polyploids: uneven and inadequate sequencing depth, copy number uncertainty, heterozygote miscalling, missing data, sequencing errors, etc., all of which challenge the integration of DNA variant information across studies^38–40^. As with the other DNA marker genotyping approaches reviewed here, the first GBS study in octoploid strawberry utilized the diploid *F. vesca* reference genome in combination with a phylogenetic approach (POLiMAPS) for aligning, classifying, and calling DNA variants^9,36^.

Recently, Hardigan et al.^11^ whole-genome shotgun (WGS) sequenced 88 *F*. × *ananassa*, 23 *F. chiloensis*, and 22 *F. virginiana* germplasm accessions. Strikingly, 80% of the short-read DNA sequences uniquely mapped to single subgenomes in the octoploid reference. Approximately, 90M putative DNA variants were identified among *F*. × *ananassa*, *F. chiloensis*, and *F. virginiana* individuals, whereas 45M putative DNA variants were identified among *F*. × *ananassa* individuals. An ultra-dense framework was then developed of genetically mapped DNA variants across the octoploid genome by WGS sequencing 182 full-sib individuals from a cross between *F*. × *ananassa* “Camarosa” and *F. chiloensis* subsp. *lucida* “Del Norte”. Large expanses of homozygosity within the commercial hybrid parent prevented complete end-to-end mapping of all 28 octoploid chromosomes in *F*. × *ananassa* as was accomplished with the wild parent, further demonstrating the value of dense NGS data for understanding sources of genotyping and mapping challenges in the octoploids. As these WGS-GBS and GBS mapping results demonstrate^10,37^, several NGS-based genotyping approaches^41–44^ should work well in combination with the octoploid reference genome^10^.

In summary, while the complexity of the octoploid genome has historically complicated DNA variant genotyping and genetic mapping in strawberry^9,14,19,24,31,45–47^, the chief technical challenges were addressed with: (a) the development of a high-quality octoploid genome assembly; (b) WGS resequencing of numerous octoploid individuals that shed light on the extent of intra- and inter-homoeologous nucleotide variation; (c) identification and physical mapping of DNA variants across the octoploid genome; and (d) comparative genetic mapping of the wild octoploid progenitors of *F*. × *ananassa* using SNPs anchored to the octoploid reference genome^10,11^.

DNA variants genotyped with different platforms and approaches predating the octoploid reference genome^9,14,31,45,47^ were independent and disconnected, resulting in the proliferation of linkage group nomenclatures, absence of a universal linkage group nomenclature, uncertainty in the completeness of genome coverage, and inability to cross-reference physical and genetic mapping information across studies, populations, and laboratories. The DNA marker sequences from many of the previously published mapping experiments were either not readily available or too short or nonspecific to enable unambiguous mapping to the octoploid reference genome^10,11^. The one exception was the genetically mapped double digest restriction-associated DNA sequence (ddRAD) markers described by Davik et al.^47^, which were used by Edger et al.^10^ for scaffolding the octoploid reference genome. Most *F. vesca* DNA probe sequences used to assay SNPs on the iStraw35 and iStraw90 SNP arrays were too short and nonspecific to unambiguously determine their physical marker locations in the octoploid genome^11^. Hence, genotypes produced with these SNP arrays could not always be effectively utilized for genome-wide association studies or other applications requiring subgenome resolution. Moreover, none of the previously published iStraw90 (or iStraw35) based genetic mapping studies have shared SNP marker genetic locations, complete genetic maps, or other critical enabling information needed to identify corresponding linkage groups across laboratories^31,32^.

These long-standing issues were resolved with the development of a new 850,000-SNP genotyping array populated exclusively with DNA variants and reference DNA sequences that unambiguously mapped to single-homoeologous chromosomes in the octoploid reference genome^11^. Using the 850,000 SNP array, a second array (“FanaSNP”) with 50,000 subgenome specific SNPs, including 5819 genetically mapped SNPs from the iStraw35 array was developed^11^ facilitating the integration of genetic and physical mapping information across studies. These new arrays provide telomere-to-telomere coverage and target common DNA variants within and among domesticated populations. Although the full set of iStraw SNP probe DNA sequences could not be unambiguously aligned to a single octoploid subgenome^11^, the true physical position for 97% of the retained iStraw probes were identified using linkage disequilibrium with the newly developed SNPs probes anchored to the octoploid reference genome^11^. Comparative mapping of SNPs in several wild and domesticated populations facilitated the integration of earlier linkage group nomenclatures and the development of a universal linkage group nomenclature substantiated by the observation of genome-wide synteny among diverse octoploid genetic backgrounds^10,11,30^.

These recent advances in genotyping and mapping are expected to have tremendous and immediate impacts on applied research in genetics and breeding of strawberry. But other research questions arise which have bearing on the utility of these new tools and resources, particularly with regard to diversity among genomes that is currently undescribed. For example, what large-scale structural variations exist in octoploid *Fragaria* germplasm? Recent advances in long read sequencing platforms (e.g., PacBio and MinION) resulted in significant decreases in costs and increases in read lengths and should soon permit inexpensive assessments of structural variants across the cultivated strawberry pangenome. On a smaller scale, what percentage of genes in cultivated strawberry exhibit presence–absence variation? Recent pangenome studies in plants have revealed that a significant proportion of gene content exhibits presence–absence variation^48–50^. For example, nearly 20% of the genes in *Brassica oleracea* are found in only certain genotypes and are enriched with functions encoding major agronomic traits. This suggests that genes in strawberry will be missed when utilizing a single octoploid reference genome and genotyping resources based on that genome alone. To construct a useful pangenome, how many individuals need to be included to capture most variation in gene content? These questions will soon be addressed as additional octoploid genomes become available.

## Genome-assisted breeding in strawberry

For many years genome-assisted breeding in strawberry lagged behind agronomic crops and even many specialty crops. However, surveys conducted by the RosBREED consortium and funded by the NIFA Specialty Crop Research Initiative have documented the rapid rise in the use of DNA information in strawberry breeding in the last decade. In 2010, only 43% of surveyed strawberry breeders had employed DNA markers or other genomics-based tools. By early 2019, data on 12 of the 14 active strawberry breeding programs in the U.S. indicated that all but one of these 12 programs (92%) had used DNA information for at least one of four purposes. The most common application was for verifying the identity or better understanding the lineage of plant materials used in the program. Two-thirds of the programs had used DNA markers or other genomics-based tools to choose parents and plan crosses, and seven of the 12 (58%) had used DNA information for seedling selection. Two-thirds of the programs were involved in upstream research of direct relevance to their programs, e.g., creating or validating DNA tests of particular applicability for their plant materials and breeding goals. Some of these were one-time or infrequent applications; however, seven of the 12 programs (59%) reported using at least one application of DNA information “on an ongoing, routine basis” (Michael T. Coe, personal communication).

Among the many breeding-relevant loci discovered in the cultivated strawberry genome, flowering, and fruit quality loci have been prominent, as would be expected in a high-value fruit commodity. These, include discovery of the locus controlling day-neutrality or PF^51^ and its subgenome localization^52^ as well as multiple loci controlling volatile compounds such as gamma decalactone, mesifurane, and methyl anthranilate^53–56^. For uncovering disease resistance loci, quantitative trait locus (QTL) mapping has been the most prominent approach. While traditional biparental populations have been effective for QTL discovery^57^, pedigree-based analysis in multiparental populations using FlexQTL^™^ has been increasingly applied^58,59^, as pedigree breeding and maintenance of clones across generations are common in strawberry. Pedigree-based analysis in complex family structures has allowed the simultaneous detection of multiple QTL alleles and the quantification of their phenotypic effects across diverse genetic backgrounds, as demonstrated for the *FaRPc2* locus^60^.

The use of DNA tests in breeding has been greatly enhanced by RosBREED efforts in marker development and validation^61^. Assays for SNP detection such as kompetitive allele-specific polymerase chain reaction (KASP) and high-resolution melting have become the tests of choice for breeding applications due to an abundance of SNP information from array genotyping, accuracy and ease of scoring, and resilience to crude strawberry DNA extracts^62,63^. The Strawberry DNA Testing Handbook was recently developed to assist breeders in identifying published DNA tests and understanding how to apply them^64^. This community resource is available at the Genome Database for Rosaceae^65^ and will be continually updated as existing tests are improved and new tests are published (Table 1).

**Table 1:** Published strawberry DNA tests included in the Strawberry DNA Testing Handbook^64^, and the loci targeted

Trait	Gene/QTL	Test ID	Platform/test type	Reference	
				Locus	DNA test
*Flowering*					
Perpetual flowering	*FaPFRU*	Bx215 SSR	SSR	Gaston et al.^51^; Verma et al.^52^	Perrotte et al.^84^; Salinas et al.^85^
*Fruit quality*					
γ-decalactone	*FaFAD1*	qFaFAD1	SCAR	Sánchez-Sevilla et al.^55^; Chambers et al.^53^	Chambers et al.^53^
γ-decalactone	*FaFAD1*	UFGDHRM5	HRM	Sánchez-Sevilla et al^55^; Chambers et al.^53^	Noh et al.^62^
Mesifurane	*FaOMT*	FaOMTSI/NO	SCAR	Zorrilla-Fontanesi et al.^124^	Zorrilla-Fontanesi et al.^124^
*Disease resistance*					
* Phytophthora* *fragariae* var. *fragariae*	*Rpf1*	SCAR-R1_A_	SCAR / SSR	Van de Weg^166^	Haymes et al.^141^;^167^ Rugienius et al.^168^; Sasnauskas et al.^169^; Mathey^170^
* Phytophthora cactorum*	*FaRPc2*-H3	RPCKASPH3	HRM / KASP	Mangandi et al.^60^	Noh et al.^63^
* Xanthomonas fragariae*	*FaRXf1*	HRM6D_33.083	HRM	Roach et al.^57^	Roach et al.^57^
* Colletotrichum acutatum*	*FaRCa1*	UFCa1HRM01	HRM	Salinas et al.^59^	Salinas et al.^171^
* C. acutatum* PG 2	*Rca2*	Rca2_240	SCAR	Denoyes-Rothan et al.^143^	Lerceteau-Köhler et al.^172^
* C. gloeosporioides*	*FaRCg1*	UFCg1HRM01	HRM	Anciro et al.^58^	Anciro et al.^58^

**Fig. 1:**
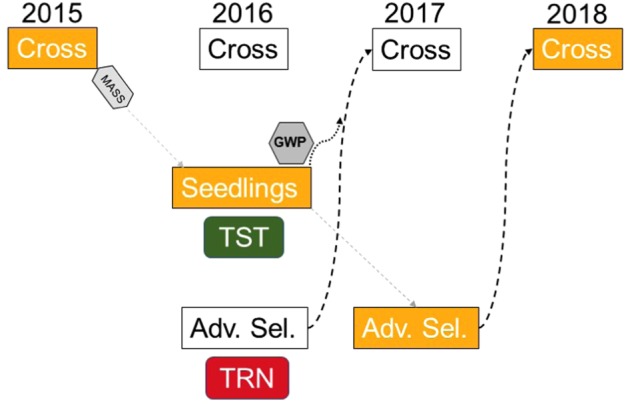
Genome-wide prediction (GWP) can reduce the strawberry breeding cycle from a minimum of 3 to 2 years. In this example, a 2016 replicated trial of advanced selections (training population) was phenotyped and genotyped and a model generated to predict parental performance (genomic estimated breeding values) for seedlings from the same year (test population) for which phenotypic data has not yet been collected. Some of the untested seedlings with high predicted performance for predicted traits of interest were used in 2017 crosses, one year before they would be used as parents without genome-wide prediction. Combining GWP for complex traits with marker-assisted seedling selection (MASS) for traits controlled by one or few genes results in a comprehensive strategy for genetic improvement

While locus-specific DNA tests are highly useful in parent and seedling selection for traits with simple genetics, genome-wide prediction has become the strategy of choice for improving genetically complex traits in crop species. The goal is predictive, and the utility of this strategy has been demonstrated in strawberry for parent selection for yield and quality traits where it was shown that: (a) markers are more effective than pedigrees for estimating breeding values, even when phenotypic information is present; (b) phenotyping effort can be reduced by using trials of advanced selections as training populations; and (c) individuals with high predicted performance can be used as parents one year early in the breeding cycle^66^ (Fig. 1).

The future of strawberry breeding research is rife with opportunities in the genomics era. In particular, candidate-gene approaches will be dramatically enhanced by the “Camarosa” reference and other octoploid genomic resources, given the ability to pinpoint sequence variations among subgenomes and thus distinguish among homoeologous alleles. Genes involved in fruit volatile compound biosynthesis are particularly attractive targets given the importance of aroma to flavor and sweetness perception^67^. Gene identification will, in turn, fuel the development of new DNA tests and enhance existing tests. Questions for the future include the following: What level of genetic gains can be achieved simply by developing markers in causal genes, eliminating problems of recombination between marker and gene? Will functional characterization of genes lead to gene edited strawberries in the commercial realm?

A tool that may help to uncover gene/trait associations in strawberry and help identify “missing heritability” is expression QTL (eQTL). In essence, eQTL are segregating genomic regions influencing differential gene expression. With RNAseq alone, it is often difficult to discern whether changes in transcript accumulation are due to genetics, environment or stochastic effects. A recent eQTL analysis identified a subset of strawberry fruit genes whose differential expression is determined by genotype, the extent of that genetic influence, and markers that can be used for selection of desired gene-expression ranges^68^. Thus, eQTL analyses may reveal marker/trait associations in cases where strawberry phenotypes are influenced by transcript abundance. In other cases, eQTL controlling transcripts of undetermined function can support candidate-gene discovery and trait-based gene cloning. In a recent example, simple cross-referencing of trait-QTL and eQTL markers identified a causal aroma biosynthesis gene in melon^69^.

For complex traits controlled by many loci, the area of genome-wide prediction presents a number of practically important research questions for the future. Will the newest SNP array, with its whole-genome coverage and wealth of subgenome-specific markers, help increase prediction accuracies? How large should training populations be to achieve maximum predictive power, and how many breeding cycles can be included? Given that the vast majority of octoploid strawberry cultivars are asexually propagated, can non-additive effects be modeled to enhance predictions of clonal performance? When will low-density genotyping be affordable enough to select for complex traits in seedling populations, as opposed to selection only among parents? These questions are important and yet are very practical and applied in nature. They have been answered in other crop species, and we expect that they will soon be answered in strawberry as well.

## The genetics of flowering, fruit development, and fruit quality traits

Because strawberry is a highly perishable fruit commodity, flowering and fruit traits are highly important from both biological and commercial standpoints. Recent genetic insights into these traits highlight several research topics of future importance.

### Flowering and runnering

Strawberries are perennial rosette plants that form a determinate inflorescence from the apical meristem of the crown. Their axillary meristems can differentiate into either branch crowns, that are able to bear additional inflorescences, or runners. Because of these alternative fates of axillary meristems, there is a strong trade-off between flowering and runnering^70,71^. Strawberries can be divided into two main groups according to their flowering habits. Seasonal flowering (SF) strawberries produce flower initials under day lengths below a critical limit (variable but often defined as <12 h), whereas perpetual flowering (PF) strawberries produce new inflorescences continuously once induced to flower.

In the diploid woodland strawberry *F. vesca*, the dominant progenitor of the octoploid cultivated strawberry^10^, two classical mutants affecting flowering and runnering are known. Recessive mutations in the SF Locus (SFL) and Runnering (R) locus cause PF and runnerless phenotypes, respectively^72^. The *F. vesca* homolog of TERMINAL FLOWER1 (FvTFL1) was found as a candidate gene for SFL independently by two groups^73,74^, and Koskela et al.^73^ demonstrated the function of FvTFL1 as a major floral repressor that causes the seasonal flowering habit. The R locus was also recently mapped, and a mutation in a gene encoding gibberellin (GA) biosynthetic enzyme GA20-oxidase (FvGA20ox4) was found. This gene is highly expressed in axillary buds, and the mutated enzyme is not able to convert GA12 to GA20, a precursor of bioactive GA1^75^.

**Fig. 2:**
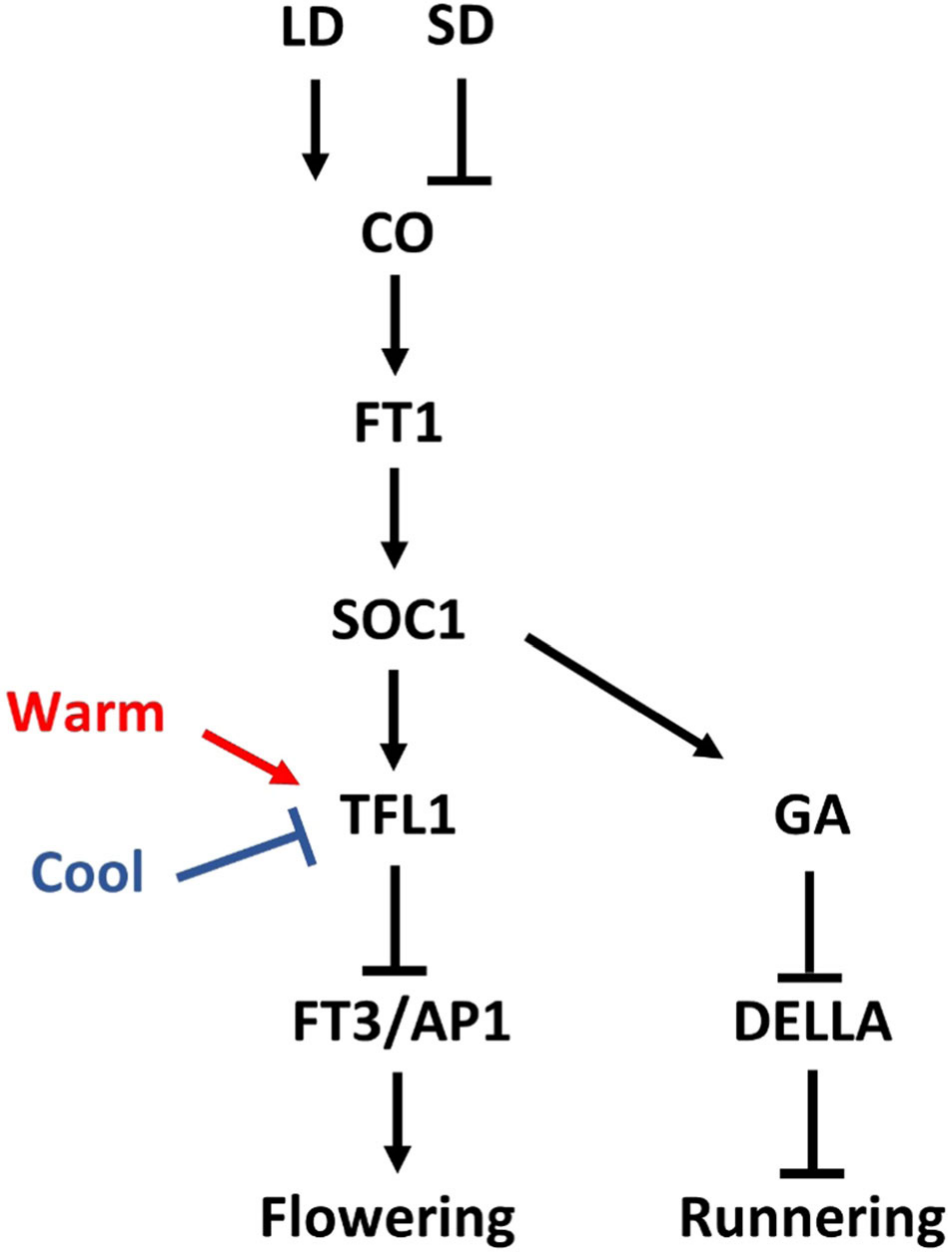
A proposed model of the regulation of flowering and runnering in strawberry. Arrows indicate activation and bars indicate repression

Studies in cultivated strawberry^76,77^ indicate at least partial conservation of the genetic pathway in woodland strawberry^73,77–80^. Based on available data in woodland strawberry, a genetic model can be proposed (Fig. 2). In SF genotypes, FvTFL1 integrates environmental signals to control flowering, and flower induction only occurs after the downregulation of this gene by cool temperatures below 13 °C or by short days at temperatures of 13–20 °C, whereas higher temperatures prevent flower induction by activating FvTFL1^79^. Genes involved in the temperature regulation of FvTFL1 await elucidation, but the photoperiodic pathway is quite well understood. Under long days, the woodland strawberry homolog of CONSTANS (FvCO) activates FLOWERING LOCUS T1 (FvFT1) in leaves, which leads to the upregulation of SUPPRESSOR OF THE OVEREXPRESSION OF CONSTANS1 (FvSOC1) in the shoot apex^78,80^. In PF woodland strawberries that are lacking a functional FvTFL1, this FvCO-FvFT1-FvSOC1 pathway promotes flowering, whereas in SF genotypes upregulation of FvTFL1 by FvSOC1 reverses the outcome of the pathway^78,80^. Actual flower induction is poorly understood, but the role of FvFT3, APETALA1 (FvAP1), and FRUITFULL (FvFUL) genes that are activated in the shoot apex after the downregulation of FvTFL1 by short days or cool temperature should be further explored^73,76,77,81^.

Another important challenge is to understand the flowering process in the context of the yearly growth cycle and to identify allelic variation that can be used for breeding new cultivars better adapted to diverse climates. Open questions include, for example, how is flower initiation and differentiation regulated? How is floral development connected to dormancy? PF cultivars are commercially quite important, but the genetic control of the trait clearly differs from PF in woodland strawberry. A major locus controlling PF was identified, named perpetual flowering and runnering (PFRU) because the PF allele also reduced runnering^51^. Several additional studies in different crossing populations have confirmed PFRU and narrowed the chromosome region^52,82–85^. The causal gene is not known, but several candidate genes have been suggested^84^. Interestingly, a QTL controlling flowering time in woodland strawberry was mapped to the same region of chromosome four^86^. These data suggest the presence of either two important flowering genes in this region or different alleles of the same gene that control PF and flowering time. Identification of the PF gene or genes in cultivated strawberry is obviously a research question of high importance both scientifically and commercially.

Better understanding the trade-off between flowering and runnering is also an important area of inquiry, because it might assist plant breeders and growers in controlling the balance between vegetative and sexual reproduction. Several lines of evidence suggest that GA controls the fate of axillary meristems in strawberries. Guttridge and Thompson^87^ found that runnerless woodland strawberry mutants began to form runners after GA treatment, and similar reversion was observed in a recent mutant screen that led to the identification of suppressor of runnerless, a gene that encodes a DELLA growth repressor of the GA signaling pathway^88^. Inhibitors of GA biosynthesis, in contrast, enhance crown branching and yield in cultivated strawberry^89,90^. Furthermore, FvSOC1 was found to control runner formation and regulate the expression of several GA biosynthetic genes, including the recently identified FvGA20ox4, which likely encodes a rate limiting enzyme of the GA biosynthetic pathway in axillary buds^75,78^. These data suggest a model in which FvSOC1 activates FvGA20ox4 and possibly other GA biosynthetic genes in axillary buds, leading to high bioactive GA1 levels, degradation of SLR proteins, and runner formation (Fig. 2).

### Fruit development

In contrast to most fruits^91^, the fleshy tissue of *Fragaria* is a modified stem tip called the receptacle. The true fruits of *Fragaria* are dried ovaries called achenes, each of which contains a single seed. The receptacle together with attached achenes are what is typically refered to as the “fruit”. Although connected by fibrovascular strands^4^, molecular analysis using microarrays^92^ and RNA-seq^93,94^ show that the achenes and receptacle exhibit asynchronous transcriptional programs that reflect differences in timing of maturation of the two tissues. Fruit set requires a sufficient percentage of fertilized achenes due to their production of auxin^95^. Early studies showed that strawberry is non-climacteric, not appearing to respond to exogenous ethylene. However, the role of ethylene in strawberry maturation is reassessed later in this section in light of more recent data.

**Fig. 3:**
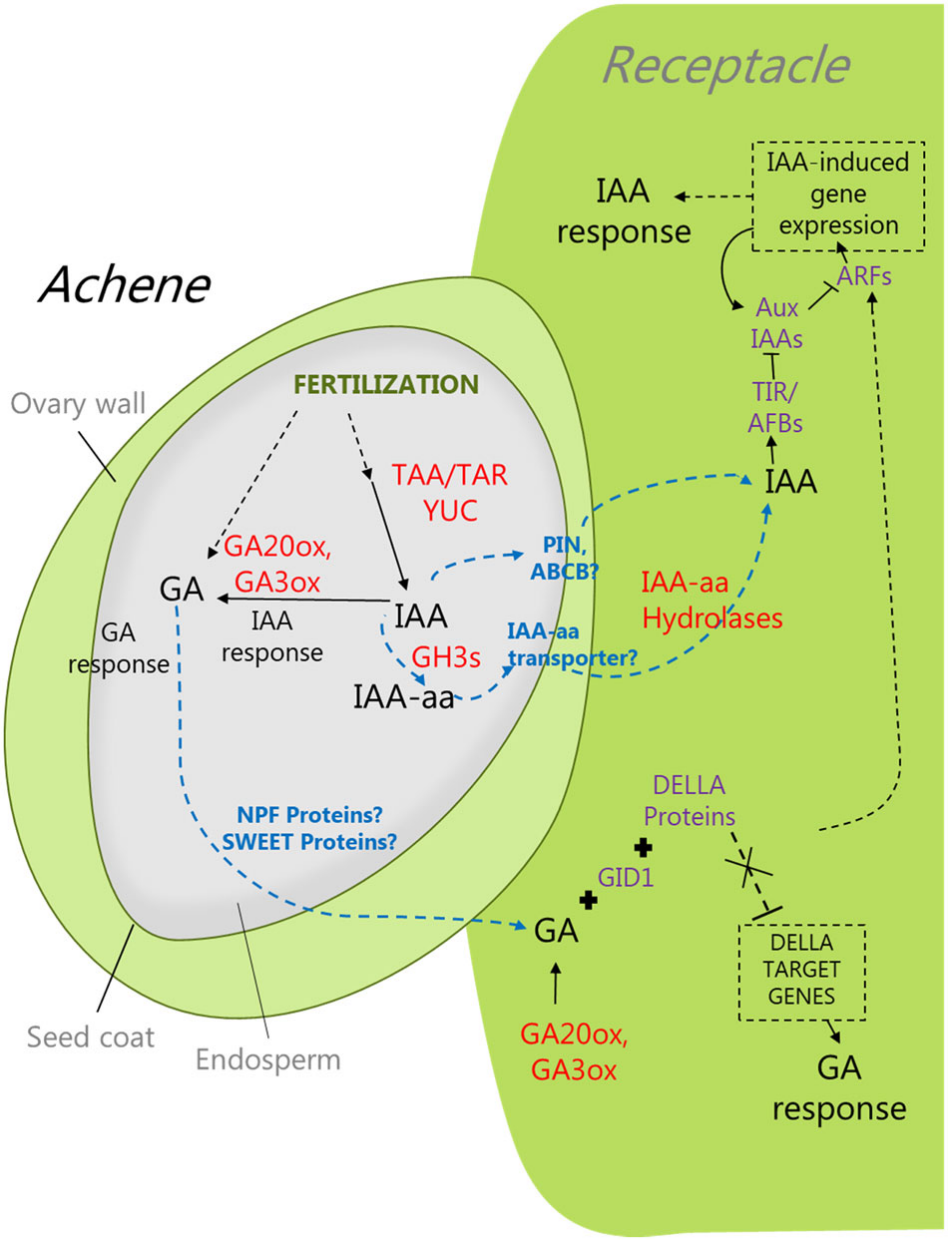
Fruit set and early fruit development set the stage for a symphony of color, flavor, and sweetness that accompanies ripening. Aspects of hormone homeostasis, transport, and signaling resulting in fruit set and early fruit development in diploid or octoploid strawberry have been implied from multiple transcriptome studies. Hormone metabolism genes are indicated in red, hormone transporters in blue, and hormone signaling components are indicated in purple. However, many question remain to be resolved (dashed lines) regarding pathway components for the two major hormones, auxin and gibberellin. These components need to be known before we can ask questions about how the genes involved in the process are regulated. Over 50 years have passed since auxin was identified as being required for receptacle enlargement, yet a fundamental question remains: what is it about fertilization that turns on the production of auxin? What form(s) of the hormones are transported from the ovule to stimulate growth of the receptacle, and what are the transporters involved? Which of the auxin and gibberellin responsive genes are critical to fruit enlargement?

The control of fruit set and development by plant hormones that interact and synchronize signals between the developing seed and surrounding tissues is graphically described in McAtee et al.^96^, and a comprehensive discussion of hormonal regulation of fruit ripening in non-climacteric as compared to climacteric fruit can be found in Cherian et al.^97^. Although a complete picture of how hormonal regulation and crosstalk underlie the molecular mechanisms of development and ripening has not yet emerged in either diploid or octoploid strawberry, considerable progress has been achieved due to gene-expression analyses using microarrays^92,98,99^, RNA-seq^93,94,100,101^, agroinfiltration for transient gene silencing, stable transformation with reporter genes or altered expression^102^, and virus-induced gene silencing (VIGS)^103^. The high-quality diploid and octoploid strawberry genomes and associated resources now available will greatly accelerate discovery. There is still much to learn from the diploid model, and fruit development in the octoploid is likely to involve a more complex interplay of homoeologous genes^104^. In addition, due to potential for interactions among products of homoeologs in the octoploid, careful holistic analysis of octoploid fruit development in achenes and during development remains to be accomplished by combining highly sensitive and accurate transcriptomic, proteomic, and metabolomic methods.

That auxin (IAA) and GAs are primary hormonal players during early development of both the receptacle and achenes is well supported by hormonal analyses of *F*. *×* *ananassa*^105^ and transcriptome data from *F. vesca*^94^. *F. vesca* transcriptome data from fertilization to the large green fruit stage (when the embryo is fully developed), were analyzed for evidence of biosynthesis and activity of most of the major hormones, as well as for IAA transport^94^. Genes in the IAA biosynthesis pathway are actively expressed by the endosperm, and perhaps integuments, of the newly fertilized ovary, closely followed by expression of GA biosynthesis genes^94^. This transcriptome foundation now needs to be expanded with detailed hormone metabolic and transport studies, perhaps more easily accomplished by feeding studies with the correct reference compounds and mass spectrometric analyses using larger octoploid fruit. As illustrated in Fig. 3, there are many steps in the process of fruit set and early development that still require direct study.

We know that IAA levels in intact *F*. *×* *ananassa* fruit rise rapidly following fertilization and peak at the small green fruit stage, thereafter decreasing to low levels at the white stage and to very low, but homeostatically regulated, levels in red ripe fruit^105^. Interestingly, separate analysis of white fruits and achenes showed that almost all the IAA measured in the intact fruit is in the achenes, with barely detectable levels in the receptacle. It has been suggested that low receptacle IAA levels are required for ABA biosynthesis to start and ripening to commence. GA_1_, likely the only bioactive GA in fruit^105^, also increased after fertilization, reaching a peak in the large green stage. Like IAA, GA_1_ levels are low during ripening stages. Analysis of spatial expression in *F. vesca* showed that genes encoding *AUX/IAA* transcriptional coregulators and auxin response factors (*ARFs*), are highly expressed in the expanding receptacle^94^, as expected if IAA is being released from the achenes to stimulate receptacle growth prior to ripening. How, and in what form, auxin travels from the fertilized achenes to the receptacle is still unknown, as are the signals that turn on auxin biosynthesis in precise tissues in response to fertilization.

Auxin transport might be most easily addressed in the larger, octoploid organs. As indicated by the diploid transcriptome studies, such analyses should include measurements of conjugated IAA biosynthesis and possible movement using mass labeled compounds. GA receptor protein genes (*FvGID1a* and *FvGID1b*) are also highly expressed in expanding *F. vesca* fruit, as are DELLA repressor genes^94^. Transcripts from genes encoding homologs of GA transporter proteins (*PTR*) were found in achenes postfertilization^94^, however, direct evidence of GA transport and responses is lacking.

Other hormones likely play roles in early fruit development, and their contributions require further study in octoploid strawberry. Cytokinin signaling genes appear to be active in young seed tissue of *F. vesca*^94^. Castasterone, a bioactive brassinosteroid (BR), was detected in young developing octoploid fruit^105^ in concordance with transcriptome data indicating biosynthesis and signaling in the receptacle of the diploid^94^. The transferase that methylates Jasmonic Acid (JA) to form the volatile compound Methyl Jasmonate was recently characterized from *F. vesca* and *F*. *×* *ananassa*^106^ but may not be important for ripening^107^. Clearly, there is a need for further investigations into the possible roles of these compounds in the developing achenes and receptacle in order to understand fruit set and growth.

Unlike what is found in climacteric fruits, experiments with *F*. *×* *ananassa* have shown that ABA is clearly a promoter of ripening in strawberry^108^. VIGS of *FaNCED1*, encoding a key enzyme in ABA biosynthesis, produced white fruits, and this phenotype was rescued by exogenous ABA^109^. RNAi mediated downregulation of an ABA receptor, *FaCHLH/ABAR* in fruits, resulted in upregulation of the negative signaling regulator *ABI1* and downregulation of positive ABA signaling regulators (*ABI3*, *ABI4*, *ABI5*, and *SnRK2*)^110^. Sucrose appears to act as a signal upstream of the ABA signaling pathway in regulating strawberry fruit ripening^110,111^.

Recently, auxin was unambiguously detected in the ripe receptacles of “Camarosa”^112^ at about the same low levels reported earlier in “Red Gauntlet”^105^. These levels are about tenfold lower than what is commonly found in leaves. However, the increase in expression of *FaTAR2* encoding the auxin biosynthesis enzyme tryptophan amino transferase, and genes encoding proteins involved in auxin perception (*FaAux/IAA11*, *FaAux/IAA14b*, and *FaAux/IAA33*) together with expression of genes involved in auxin signaling (*FaARF6a* and *FaARF16c*) in ripening receptacles strongly suggests cell-autonomous auxin synthesis and cell-specific response in the receptacle at ripening^112^. Laser capture microdissection and newer methodologies in mass spectral analysis of very small amounts of tissues^113^, for example, are needed to specify which types of cells (cortex, pith, and vasculature) are engaged in hormone metabolism in the ripening receptacle.

The critical importance of studying development and ripening in achene and receptacle separately is discussed in Merchante et al.^114^, who found that in *F*. *×* *ananassa*, expression of ethylene biosynthesis gene families (*ACC SYNTHASE* and *ACC-OXIDASE*) was temporally and organ specific, and this applied as well to which members of a given gene family were expressed. Their results support many earlier reports implicating ethylene as playing a role in strawberry ripening. The most recent support for a role for ethylene in strawberry ripening comes from global analysis of transcriptomic changes in the achene and receptacle during ripening^100^. Analysis of the *FaERF* gene family identified three members, *FaERF3*, *FaERF6*, and *FaERF71a*, that are significantly expressed in the receptacle and upregulated upon ripening. Downregulating *FaSAMS1* or *FaCTR1* using the VIGS technique in the receptacle inhibited fruit red color formation. Ethephon application promoted natural red color development in white (VIGS) fruits and partially rescued FaSAM1-RNAi and FaCTR1-RNAi fruit. The results implicate *FaCTR1* as a positive regulator and ethylene as a required signaling molecule in strawberry fruit ripening. Therefore, ethylene appears to be required for the normal development of the strawberry fruit, where it acts differently in the achenes and the receptacle. In achenes, it acts at the green and red stages, while in the receptacle it acts at the green/white stages. In these organs, ethylene selectively appears to influence the expression of genes involved in ethylene reception, phenylpropanoid metabolism, cell wall degradation, and strawberry aroma production.

The expression pattern of the gene encoding the BR receptor, *FaBRI1*, in *F*. *×* *ananassa* receptacle suggests that BR may also play a role in ripening, and VIGS of this gene results in failure to redden^115^. However, a direct role for this class of hormone in strawberry ripening is in question, as neither active BR castasterone nor brassinolide were detected by the end of the white stage using unambiguous and sensitive analytical techniques^105^.

Epigenomic aspects of strawberry fruit ripening also deserve future consideration. Transcriptional regulation of fruit ripening in tomato is well characterized^116^, and although strawberry is non-climacteric it is likely that there are conserved pathways and regulatory mechanisms in common with the ripening achene or ripening receptacle. In tomato fruit, chromatin remodeling activities as well as changes in DNA methylation influence normal ripening in maturing fruit tissues^116^. Such studies in strawberry lag well behind. Recently, 71 genes encoding enzymes responsible for histone lysine methylation modifications were identified and characterized in the *F. vesca* genome^117^. qPCR showed that, in the receptacle, expression of some of the SET methyltransferase genes peak at turning stage; an indication of a role for chromatin remodeling in strawberry fruit ripening. In addition, nine DNA methyltransferase genes and four demethylase genes were identified in the *F. vesca* genome^118^. These reports indicate that DNA methylation changes dramatically at the onset of ripening, warranting a detailed investigation of the roles of epigenomics in development and ripening in octoploid fruit.

Recent comparative transcriptome analysis of developing fruit of two wild selections of *F. pentaphylla* that differ in ripe fruit color (white vs. red) demonstrated a key role of long noncoding RNAs (lnRNAs) in fruit development and fruit color formation^119^. Future studies must elucidate the functions of the genes targeted by these differentially expressed lnRNAs and their roles in the cultivated strawberry. Superimposing transcriptomics, proteomics, and metabolomics in the same tissues will allow for a more precise determination of how fruit set, development, and ripening are regulated, pointing to the most productive areas for genetic manipulation to improve fruit growth and quality.

### Fruit quality

External quality characteristics of ripe strawberry fruit including size, color, and absence of surface defects have always been a focus of research and genetic improvement. In the last decade, flavor has gained increased importance as a quality attribute demanded by consumers. Strawberry flavor is imparted by sugars (primarily glucose, fructose, and sucrose), acids (citric and malic acids), and an unknown number of over 360 reported volatiles. A comprehensive study, using psychophysics to determine attributes that influence pleasure and sensory perception of strawberry fruit, found that overall liking was most greatly influenced by sweetness and strawberry flavor intensity^67^, which are affected by environmental pressures that reduce sucrose and total volatile content. While sucrose was the single metabolite with the most significant contribution to overall liking, it was found that volatiles influence perception of both flavor and sweetness through retronasal olfaction^67^. Thirty-eight volatile compounds significantly enhanced the perceived intensity of sweetness and may be worthwhile targets for molecular study^67^. Four of these volatiles are common to most studies: 3,7-dimethyl-1,6-octadien-3-ol (linalool); the methyl and ethyl esters of butanoic acid; and 2,5-dimethyl-4-methoxy-3(2H)-furanone (mesifurane). Linalool imparts a sweet, floral, citrus-like note to strawberries, while the closely related terpene nerolidol imparts a rose/apple/green note. Mesifurane is said to have a sherry-like or fusty aroma, while its precursor, furaneol (2,5-dimethyl-4-hydroxy-3(2 H)-furanone) imparts caramel and sweet notes at high concentrations. How confident can we be in these results, and are there other fruit compounds with measurable effects on consumer perception and liking? Further research combining sensory and fruit chemical analyses, including more germplasm and environments, would be valuable for answering these questions.

Several strawberry genes involved with production of compounds contributing to flavor/aroma were identified relatively early, including *FaSAAT*, encoding a fruit-specific ALCOHOL ACYLTRANSFERASE that is exclusively expressed in receptacle tissue^120^; *FaOMT*, encoding an O-methyltransferase catalyzing the formation of mesifurane from furaneol^121^; and *FaNES1* encoding a nerolidol synthase capable of generating linalool or nerolidol with geranyl diphosphate or farnesyl diphosphate, respectively, as substrates^122^. *FaNES1* is present and highly expressed in the fruit of 112 *F*. *×* *ananassa* cultivars as well as in all but three of 46 octoploid wild *F. virginiana* and *F. chiloensis* progenitor species accessions^123^. Conversely, *FaNES1* was not present in any diploid, tetraploid, or hexaploid accession tested. Instead, the olefinic monoterpenes, namely, α-pinene, β-phellandrene, and β-myrcene are produced, which contribute to turpentine-like, woody, resinous, and unpleasant odors that are selected against by commercial strawberry breeders^123^.

QTL analysis in a population segregating for production of mesifurane and other volatiles identified a homoeolog of *FaOMT* as the locus responsible for natural variation of mesifurane content^124^. Mesifurane nonproducers lack a 30 bp promoter sequence containing putative binding sites for basic/helix–loop–helix, MYB and BZIP transcription factors. This polymorphism fully cosegregates with both the presence of mesifurane and the high expression of *FaOMT* during ripening^124^.

The amount of the volatile γ-decalactone, which is associated with “peach-like” aroma in strawberry fruit, is highly environmentally influenced. Using a metabolomics approach combined with RNAseq, Chambers et al.^53^ identified the fatty acid desaturase gene (*FaFAD1*) essential to its biosynthesis. In parallel, Sánchez-Sevilla et al.^55^ discovered the same locus by combining transcriptome analysis with a map-based approach. Interestingly, about half of cultivars tested were nonproducers of this volatile and had a deletion of this gene, pointing to *FaFAD1* as a potential target for breeding or engineering back into desirable cultivars. Methyl anthranilate (MA) contributes to the fruity, flowery, and aromatic flavor of the woodland strawberry, *F. vesca*. MA was only found in a few old strawberry cultivars such as “Mieze Schindler” and “Mara des Bois”^125^, and a gene encoding ANTHRANILIC ACID METHYL TRANSFERASE (FanAAMT) was recently isolated from the latter using transcriptome bulk-segregant analysis^54^. While FanAAMT modulates the amplitude of MA accumulation, additional genes hypothesized to be required for basal MA production have yet to be identified. Consumer preference and yield penalties or advantages will help determine the balance of volatiles in cultivars of the future. The availability of high-quality octoploid and diploid strawberry genomes opens opportunities to manipulate known genes involved in volatile production and identify genes required for production of the many other volatiles involved in strawberry aroma.

Texture is another strawberry fruit quality attribute of great importance, both for consumer sensory preference and quality after cold storage. Several strawberry genes encoding enzymes involved in the disassembly of fruit cell walls and the solubilization of pectins in the middle lamella that results in softening during fruit ripening have been identified. In the past 5 years, genes encoding pectin solubilizing enzymes: pectate lyase (*FaplC*); endo-β-1,4-glucanase (*FaEG3*)^126^; β-galactosidase (*FaβGal1*)^127^; and polygalacturonase (*FaPG1*)^128^ have been implicated in fruit softening during ripening. Nardi et al.^129,130^ have demonstrated a role for expansins, the nonenzymatic cell wall proteins that are associated with cell wall loosening, in strawberry ripening by functional analysis of the promoter of Expansin 2 (*FaEXP2*). Recently, the xyloglucan endotransglycolase/hydrolase gene family was characterized in *F. vesca* (*FveXTHs*)^131^. These enzymes modify xyloglucans that cross-link cellulose microfibrils. Most of the 26 genes identified are expressed in fruit, but expression of a subset of four genes increases during fruit softening. Fruit texture affects not only sensory perception, but also the ability of strawberry to withstand packing and long distance shipping without bruising. Additional information regarding the identity and regulation of genes encoding enzymes that contribute to fruit softening in strawberry can be mined from the existing *Fragaria* transcriptome data, and their function(s) analyzed using stable transformation, transient expression, and CRISPR technology. Cultivars or wild accessions with the desired firmness qualities that satisfy producers and consumers could help mitigate fruit losses postharvest.

Not to be ignored is the role of small noncoding RNAs such as microRNAs (miRNAs) in fruit quality, since these are known effectors of regulatory pathways underlying plant development including fruit ripening. In tomato, miRNAs were differentially expressed in the developing fruit, and one miRNA, sly-miR1917, targets a transcription factor that negatively regulates ethylene responses during ripening^132^. Almost 200 miRNAs were identified in *F*. *×* *ananassa*^133,134^. In *F. vesca*, one of these, miR399, appears to be involved in soluble solids content and in fructose and glucose content^135^. The molecular mechanism of miRNA399 action is not known and roles of miRNAs in regulating various aspects of *F*. × *ananassa* fruit quality require further investigation.

Other important quality factors include fruit color and phytochemical compounds important to human health. FaMYB10 is one of the known transcription factors involved in *F*. *×* *ananassa* fruit ripening (Table 2).

**Table 2:** Known transcription factors involved in octoploid *Fragaria* fruit ripening

TF type	Gene name	Accession no.	Function	Reference
AP2/ERF	*FaABI4*	MH332931.1	Positive regulator of ripening	Chai and Shen^173^
R_2_R_3_ MYBS	*FaMYB1*	AAK84064.1	Repression of anthocyanin biosynthesis	Aharoni et al.^174^; Kadomura-Ishikawa et al.^175^
	*FcMYB1*	ADK56163.1	Anthocyanin production in *F. chiloensis*	Salvatierra et al.^176^
	*FaMYB10*	ABX79947.1	Master regulator of flavonoid/phenylpropanoid metabolism. Expression repressed by auxin, stimulated by ABA.	Medina-Puche et al.^177^; Lin-Wang et al.^178^; Kadomura-Ishikawa et al.^179^
	*FaMYB9*	AFL02460.1	Proanthocyanidin biosynthesis in complex with FaMYB9/FaMYB11, FabHLH3, and FaTTG1	Schaart et al.^180^
	*FaMYB11*	AFL02461.1	Proanthocyanidin biosynthesis in complex with FaMYB9/FaMYB11, FabHLH3, and FaTTG1	Schaart et al.^180^
	*FaGAMYB*		Production of anthocyanins and hydroxycinnamic acid derivatives needed for eugenol production	Vallarino et al.^181^
	*FaEOBII*	AJZ73158.1	Regulation of eugenol biosynthesis under control of MYB10	Medina-Puche et al.^98^
	*FaPCL1-like*		Flavonol synthesis	Pillet et al.^182^
c-type MADS-box	*FaSHP*	AGU92563.1	TAGL1 homolog. Expression of ripening related genes	Daminato et al.^183^
e-type MADS box	*FaMADS1a*		SEPALLATA type. IAA induced expression. Delayed ripening.	Lu et al.^184^
	*FaMADS9*		SEPALLATA type. Proanthocyanidin biosynthesis with FaMYB11, FabHLH3, and FaTTG1	Seymour et al.^185^
ZnF-DOF (one zinc finger)	*FaDOF2*	AIZ50709.1	Eugenol production in ripe receptacles, interacting with *FaEOBII*	Molina-Hidalgo et al.^186^
basic Helix-Loop-Helix	*FabHLH3*	AFL02463.1	Proanthocyanidin biosynthesis in complex with FaMYB9/FaMYB11, FabHLH3, and FaTTG1	Schaart et al.^180^
WD40 repeat	*FaTTG1*	AFL02466.1	Coordinates complex with FaMYB9/FaMYB11, FabHLH3, and FaTTG1 to regulate proanthocyanidin biosynthesis	Schaart et al.^180^
GRAS	*FaSCL8*		Expression of MYB10, MYB9, and MYB11	Pillet et al.^182^
GARP	*FaPCL1-like*		Flavon-3-ol production	Pillet et al.^182^

Transcriptome analyses have identified many other transcription factors likely to be involved in strawberry ripening in diploid and octoploid strawberry. Only those with direct evidence for involvement in octoploid strawberry ripening are presented here

This transcription factor is the master regulator of flavonoid/phenylpropanoid metabolism resulting in the red color of the dessert strawberry. Throughout the world consumer preferences vary, with some preferring deeper, more purple coloration, and others a brighter or lighter red. Likewise, preferences for fruit shape differ. Some producers are now promoting a small cigar-shaped fruit rather than fruit with the familiar heart-shape because of ease of packaging. Recently reported results implicate auxin and GA in fruit width and length, respectively, in *F. vesca*^136^. The molecular underpinnings of color variation and fruit shape in *Fragaria* are mostly unknown or unreported, although clearly of interest for development of molecular markers for breeding purposes to meet changing consumer tastes.

In strawberry, antioxidant compounds such as polyphenols and ascorbic acid (vitamin C) are important nutritional traits^137,138^. Yet these are difficult traits to assess as they are influenced not only by genotype, but by the growing environment and by developmental stage. For example, levels of the bioactive nonflavonoid polyphenol, ellagic acid (EA), is higher in achenes from ripe fruit of the *F. vesca* cultivar Yellow Wonder than in achenes from ripe fruit of *F*. *×* *ananassa* cultivar Calypso. A complication for improving fruit nutrient quality is that EA levels are higher in achenes than in receptacles of all cultivars tested, and EA is found primarily at small green stage. In addition, the mode of inheritance of EA content is yet to be elucidated^139^. Previously unidentified bioactive compounds, such as the acylphloroglucinol glucosides discovered in *F*. *×* *ananassa* while examining the enzymatic properties of recombinant *F. vesca* chalcone synthases^140^, may also exist in *Fragaria* species. With the availability of modern methods in metabolomics and allied fields, discovery of additional bioactive compounds is likely, and these methods can be applied to direct molecular approaches to improving fruit quality. In the future, metabolic flux analysis should also enhance our ability to delineate what biochemical pathways are good targets for fruit quality improvement as well and to predict what modifications may influence fruit quality parameters.

## Plant–pathogen interactions

There has been ample progress in recent years in describing the genetic architecture of disease resistance in cultivated strawberry. Many resistances appear to be primarily conferred by one or two major loci or large-effect QTL, including to *Phytophthora fragariae*^141^*, Xanthomonas fragariae*^57^*, Phytophthora cactorum*^60^, *Fusarium oxysporum f.sp fragariae*^142^, *Colletotrichum gloeosporoides*^58^, and *Colletotrichum* *acutatum*^59,143^. For *P. cactorum*, additional minor loci have recently been identified^144^. On the other hand, resistances to *Verticillium dahaliae*^145^ and *Podosphaera aphanis*^146^ appear to be quite complex, with no major loci identified to date. This suggests that genomic prediction approaches for these two diseases would be most effective. However, with the advent of the “Camarosa” genome, an opportunity exists to characterize Mildew Locus O (MLO) genes in strawberry toward potential gene editing solutions.

The genetic architecture of resistance to charcoal rot (*Macrophomina phaseolina*) has not yet been reported in strawberry. Elucidating the genetics of resistance to *M. phaseolina* should be a high priority in the future, given the recent spread of this pathogen in important production regions and the lack of effective controls for this disease^147^. In addition, no resistance genes have been reported against gray mold caused by *Botrytis cinerea*^148^. Instead, it seems most likely that any small differences in tolerance to this disease among cultivars results from morphological variations in flower structures, fruit firmness, etc. Because strong resistance to *B. cinerea* is not likely to result from conventional breeding, a gene editing solution may be most viable.

Where disease resistances are conferred by one or a few genes, genetic and breeding approaches to characterize and increase resistance are straightforward. In the cases where classical R genes are involved, the development of custom-capture libraries and single-molecule resequencing of captured target sequences has been quite effective for identifying causal gene variants^149^. In fact, such a resource has now been developed for cultivated strawberry in the form of a RenSeq library based on the “Camarosa” reference and resequencing of a number of elite cultivars and breeding lines^150^. Combining this resource with mapping and association genetics approaches should help uncover subgenome-specific variants underlying known loci and lead to the cloning of R genes in octoploid strawberry. Given the tremendous allelic diversity present in strawberry and the large copy numbers and highly repetitive coding sequences typical of R genes, assembling long reads from single-molecule real-time sequencing should be helpful to this endeavor.

Hand in hand with characterization of R genes, we recommend the characterization of pathogen populations in order to understand the durability of resistances. The paradigm of a gene-for-gene arms race has been long established, but a more accurate assessment of the durability of resistance could arise from an understanding of the selective forces operating on pathogen effectors. Dual RNA-seq technology can help uncover the dynamic interactions of pathogen and host^151–153^. Both the pathogen and the host transcriptomes are simultaneously captured and analyzed in silico to distinguish species-specific transcripts. For some complex interactions, single cell transcriptomics coupled with protein and metabolite analysis may be helpful.

What new insights into disease resistance in strawberry could be gained simply from studying the population structures of causal pathogens? Would identifying and characterizing pathogen effectors give us meaningful insights into the control of pathogens through breeding and other means? It is intriguing that some recently discovered resistance loci in strawberry confer very strong resistances and yet have apparently been durably effective in commercial production for many decades^59,142^. Cloning the first R genes and pathogen effectors involved these interactions will help us to understand why.

## The Genome Database For Rosaceae: a vital resource for strawberry research

The Genome Database for Rosaceae (GDR, https://www.rosaceae.org)^154^ is the central repository and data-mining resource for genomics, genetics, and breeding data of Rosaceae, including strawberry and related crops such as almond, apple, apricot, blackberry, cherry, peach, pear, plum, raspberry, and rose. The volume and type of data generated for strawberry research has markedly increased in the past ten years. This includes whole-genome assembly data, RNA-seq data, multiple SNP arrays, increased numbers of QTL, and more genotypic and phenotypic data. The massive volume of data generated by the strawberry research community, combined with active curation, integration, further analyses and tool development by the GDR team has resulted in marked expansion in the data and functionality available for strawberry.

In addition to the near-complete chromosome-scale assembly for *F*. *×* *ananassa*^10^, two draft genome assemblies for *F*. *×* *ananassa*^155^ are available. Four genome assemblies, including the newest v4.0^30^, are also available for *F. vesca*. New and much improved annotation v4.0.a2^156^, including 34,007 protein-coding genes with 98.1% complete Benchmarking Universal Single-Copy Orthologs (BUSCOs), is available. For older assemblies *F. vesca* genome v1.1^157^ and v2.0^9^, additional annotations are also available: v1.1.a2^158^ and v2.0.a2^156^, respectively. The draft genome assemblies of four wild diploid *Fragaria* species^155^ and of *Potentilla micrantha*^159^ a species that does not develop fleshy fruit but is closely related to *Fragaria*, are also available. In addition, the whole genome of *F. iinumae*^21^ has recently become available.

GDR now provides a reference transcriptome (*F*. *×* *ananassa* RefTrans V1) that combines published RNA-Seq and EST data sets. The GDR team provides additional computational annotation for both predicted genes of whole-genome assemblies and RefTran datasets with homology to genes of closely related or model plant species and assignment of InterPro protein domains^160^ and GO terms^161,162^. The genome assembly and transcript data can be accessed through the *Fragaria* genus and species pages, Gene/Transcript search page, JBrowse^163^ and BLASTX^164^.

The octoploid “Camarosa” genome, *F. iinumae* v1.0, and both annotation versions of *F. vesca* Genome v4.0, are used in a synteny analysis with whole-genome assemblies from 18 Rosaceae species using MCScanX^165^ with results available to view and search through the Synteny Viewer. GDR hosts 29 genetic maps for *Fragaria* species, most of which contain trait loci and can be viewed and compared through the MapViewer. Detailed data on 505 QTLs and 5 MTLs for 124 horticultural traits, and 171,115 genetic markers for *Fragaria* that includes 154,739 SNPs are available, as well as SNP data from the iStraw 90 K array for cultivated strawberry^32^. The SNP data is accessible through JBrowse tracks, downloadable files and can be searched and downloaded from the SNP Marker and All Marker search pages. The Marker search page now includes filtering by trait name, which allows users to search for markers that are near and/or within QTLs using the associated trait name. Phenotyping data from the public projects such as RosBREED^61^ are available from GDR. In addition to the “Search Trait Evaluation” page, the public breeding data can be queried and downloaded using the Breeders toolbox. A new module in GDR, the Breeding Information Management System (BIMS), now provides breeders and breeding project teams with tools to easily store, manage, archive and analyze their private or public breeding data.

The availability of whole-genome assembly and SNP array data for the cultivated octoploid strawberry, along with wealth of QTL data that are integrated in the community database with data from other related crops are expected to accelerate research and practical tools such as DNA tests. BIMS in GDR will help breeders not only to organize their data but also to utilize the tools and resources that are available for strawberry improvement.

## Conclusions

**Fig. 4:**
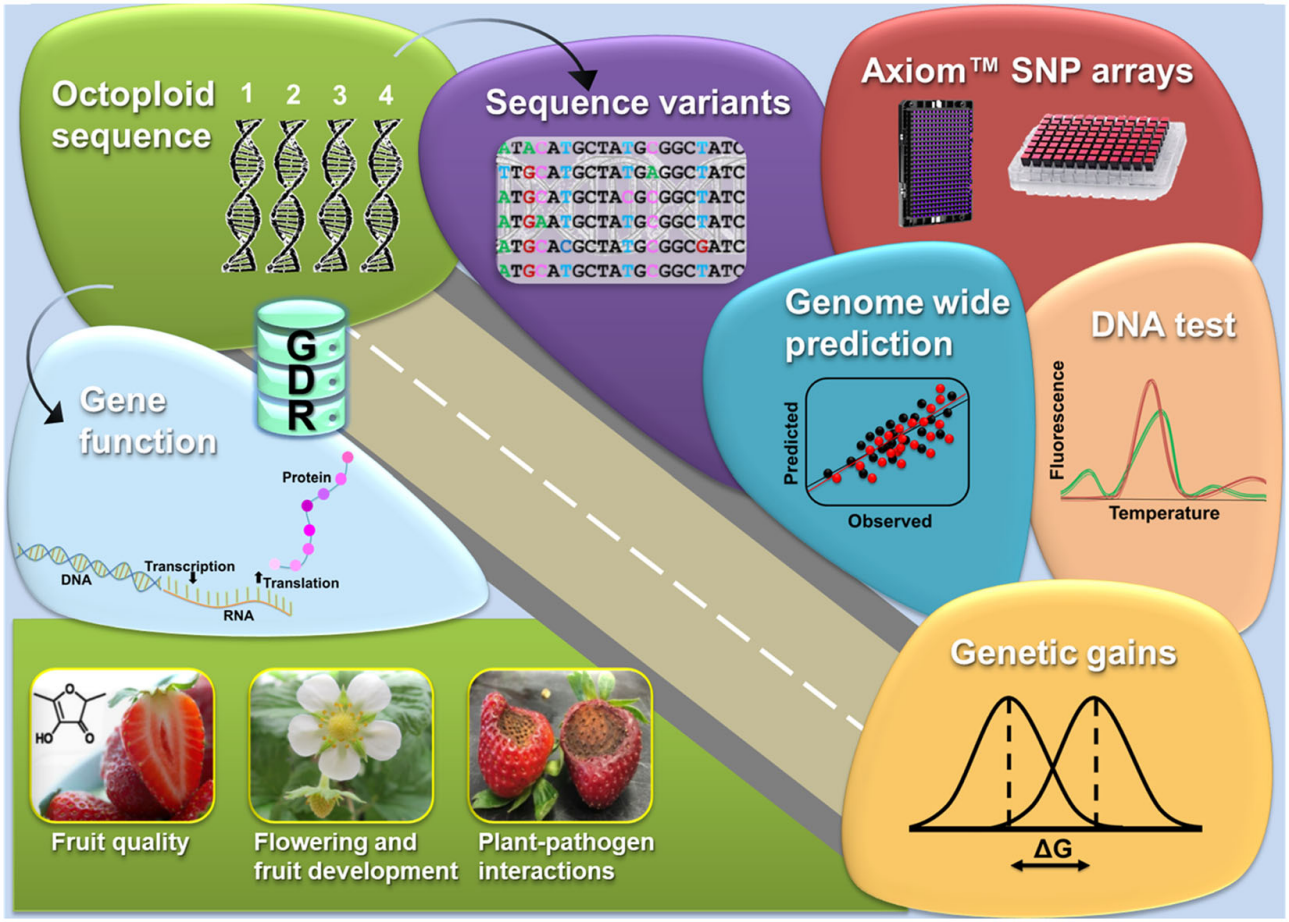
A roadmap for future research in octoploid strawberry that begins with high-quality genome resources and leads to genetic improvement, through both basic and applied avenues

The first high-quality chromosome-scale assembly of cultivated strawberry is the starting point for an exciting research roadmap for the future (Fig. 4). The debate over the origins of the octoploid genome will continue to evolve. Meanwhile, the new “FanaSNP” array will enable genetic and breeding advances, including increased DNA test availability. We expect that the use of genomic prediction for complex traits will rapidly increase, in proportion to the increased quality of array genotyping in strawberry and advances in prediction methods. Basic research on gene function will accelerate discoveries on the biology of many phenotypes, especially fruit development, fruit quality and plant-pathogen interaction traits. In particular, advances in understanding the biosynthesis of aromatic volatile compounds could help form the basis for more flavorful strawberries. One of the most exciting prospects is the development of an octoploid pangenome that reveals the DNA variation underlying the striking phenotypic variability in cultivated strawberry and its immediate progenitors. In order to maximize these opportunities, it is critical that the strawberry research community continue to invest in the Genome Database for Rosaceae and other collaborative genomic resources and endeavors at a global scale.
